# The Yellow Fever Experiments in Havanna

**Published:** 1901-10-05

**Authors:** 


### THE YELLOW FEVER EXPERIMENTS IN HAVANNA.

However ready we may be to admit the necessity of experiment in the investigation of pathological problems, we cannot believe that the sanction of the profession at large will be accorded to the methods which, according to recent reports, have been adopted in Havanna with the object of investigating the manner in which the infection of yellow fever is distributed and of ascertaining the efficacy of certain vaunted remedies for the disease. We refer more particularly to experiments on human beings.

For some time past evidence has been accumulating to show that the mosquito is at least one of the means by which the infection of yellow fever is distributed. It has been shown again and again that mosquitos which have been fed upon yellow fever patients are able to infect others with this disease, and it has been sought, apparently with some success, to convey, by means of the mosquito, an infection of such a mild or attenuated character as by degrees to confer immunity to the more virulent disease—a process analogous to the old method of inoculation against smallpox.

Against any such proceeding as this we say nothing. Yellow fever is a very deadly disease, and for the sake of obtaining protection from its attacks it is worth while running a certain risk. Anyone who submits to such treatment balances the immediate danger of the inoculation against the well-recognised risks of the disease, and he undergoes the process for the very purpose of protecting himself against the fever which prevails around him. When, however, men submit to inoculation not for the sake of the protection to be gained by it, but in return for a mere money payment—when, in other words, men sell their living bodies for experiment—we think it time to protest. This is just what seems to have occurred in Havanna, with the additional abomination that it was all part of a scheme for exploiting a secret remedy by a commercial company.

According to the statement of Major Havard, M.D., Chief Surgeon at Havanna, a Dr. Caldas and his assistant, together with a "business agent," arrived in Havanna and gave out that he had discovered the pathogenic microbe of yellow fever; had prepared a "curative serum" as well as a prophylactic vaccine, both of which had been thoroughly tested, and desired the opportunity of demonstrating their efficacy before an official commission. After some correspondence between the Acting Military Governor and the War Office, a thoroughly good Commission of yellow fever experts was appointed to examine the claims set forth.

In the course of its proceedings this Commission not unnaturally wished Dr. Caldas to demonstrate his microbe and describe the method of its isolation and culture. This, however, "he formally declined to do, because of his pledge to the company organised to exploit his discovery," and the Commission, in the end, decided to decline all active co-operation in his tests. It recommended, however, that the necessary facilities should be afforded to him, and the Commission "stood ready, in its official capacity, to watch results and report upon them." The result was this, that "four non-immune immigrants" were engaged and entered into a written contract whereby "they voluntarily offered themselves to be inoculated with Dr. Caldas' vaccine, and then to be bitten by infected mosquitos, for a certain stipulated sum of money to be paid on the termination of the experiment." Two of these men were strongly suspected by the Commission to be immune, and were rejected. The other two, "young, robust, recently-landed Spaniards," were injected with the serum and inoculated with the protective vaccine, and then in due season one of them was bitten by a pair of infected mosquitos. These two mosquitos had been hatched in a glass jar, and, therefore, were free from any suspicion of contamination until, on July 20th, they were applied to the arm of a yellow fever patient, and thus infected. On August 13th one of them was permitted to bite a young American, who within three days developed a mild attack of yellow fever. The other was one of two mosquitos which on August 14th, at her own desire, bit an American female nurse, who four days afterwards developed a very grave attack of yellow fever, from which she died. The outcome was that the young Spaniard also died. But even if he had lived we should still question the morality of healthy people selling themselves for experimental purposes.

